# ﻿Comparative mitogenomics of the genus *Motacilla* (Aves, Passeriformes) and its phylogenetic implications

**DOI:** 10.3897/zookeys.1109.81125

**Published:** 2022-07-01

**Authors:** Chao Yang, Xiaojuan Du, Yuxin Liu, Hao Yuan, Qingxiong Wang, Xiang Hou, Huisheng Gong, Yan Wang, Yuan Huang, Xuejuan Li, Haiyan Ye

**Affiliations:** 1 College of Life Sciences, Shaanxi Normal University, Xi’an 710062, China Shaanxi Institute of Zoology Xi’an China; 2 Shaanxi Institute of Zoology, Xi’an 710032, China Shaanxi Normal University Xi’an China; 3 School of Basic Medical Sciences, Xi’an Medical University, Xi’an, China Xi’an Medical University Xi’an China

**Keywords:** Comparative analysis, mitogenome, phylogeny

## Abstract

The genus *Motacilla* belongs to Motacillidae (Passeriformes), where mitochondrial features are poorly understood and phylogeny is controversial. Whole mitochondrial genome (mitogenome) data and large taxon sampling are considered to be ideal strategies to obtain this information. We generated four complete mitogenomes of *M.flava*, *M.cinerea*, *M.alba* and *Dendronanthusindicus*, and made comparative analyses of *Motacilla* species combined with mitogenome data from GenBank, and then reconstructed phylogenetic trees based on 37 mitochondrial genes. The mitogenomes of four mitogenome sequences exhibited the same gene order observed in most Passeriformes species. Comparative analyses were performed among all six sampled *Motacilla* mitogenomes. The complete mitogenomes showed A-skew and C-skew. Most protein-coding genes (PCGs) start with an ATG codon and terminate with a TAA codon. The secondary structures of RNAs were similar among *Motacilla* and *Dendronanthus*. All tRNAs except for trnS(agy) could be folded into classic clover-leaf structures. Three domains and several conserved boxes were detected. Phylogenetic analysis of 90 mitogenomes of Passeriformes using maximum likelihood (ML) and Bayesian inference (BI) revealed that *Motacilla* was a monophyletic group. Among *Motacilla* species, *M.flava* and *M.tschutschensis* showed closer relationships, and *M.cinerea* was located in a basal position within *Motacilla*. These data provide important information for better understanding the mitogenomic characteristics and phylogeny of *Motacilla*.

## ﻿Introduction

In most animals, the mitochondrial genome (mitogenome) contains 13 protein-coding genes (PCGs), two rRNA genes (rRNAs), 22 tRNA genes (tRNAs), and one noncoding region (the control region, CR) (Wolstenholme 1992; Boore 1999). Mitochondrial sequences are commonly used for inferring phylogeny (Hassanin et al. 2005), and the mitogenome has been used as an effective marker for exploring the phylogenies of some avian taxa (Li et al. 2016a; Mackiewicz et al. 2019; Cai et al. 2019).

Passeriformes comprises 6533 currently described species (Gill et al. 2020). The genus *Motacilla* belongs to Motacillidae (Passeriformes) and contains 12 species (Alström et al. 2003; del Hoyo et al. 2004), which show striking plumage pattern variation (Harris et al. 2018). *Motacillaflava* Linnaeus, 1758 is a small, insectivorous oscine (Ödeen and Björklund 2003) and is closely related to *M.alba* Linnaeus, 1758, distributed in the Palearctic (Dong and Zhang 2011). Some mitochondrial fragments, such as nad2 and CR of *M.alba* (Li et al. 2016b), have been used to study the phylogeography and population history of *Motacilla*. Additionally, some mitochondrial genes, such as nad2 (Suppl. material 1: Fig.S1A; Dong et al. 2016) and cytb (Suppl. material 1: Fig. S1B; Zhang et al. 2016), have been used to study the phylogenetic relationships of *Motacilla*. However, the phylogenetic position of some *Motacilla* species is still controversial. For example, *M.alba* has been reported to form a sister group with *M.madaraspatensis* Gmelin, 1789 (Suppl. material 1: Fig. S1A, Dong et al. 2016), but it has also been grouped with *M.cinerea* Tunstall, 1771 (Suppl. material 1: Fig. S1B; Zhang et al. 2016). In addition, phylogenetic results reconstructed from genome-wide SNPs (Suppl. material 1: Fig. S1C, Harris et al. 2018) have some incongruence with those based on mitochondrial genes or mitogenomes (Suppl. material 1: Fig. S1A, B, D; Dong et al. 2016; Zhang et al. 2016; Gao et al. 2019).

**Figure 1.**
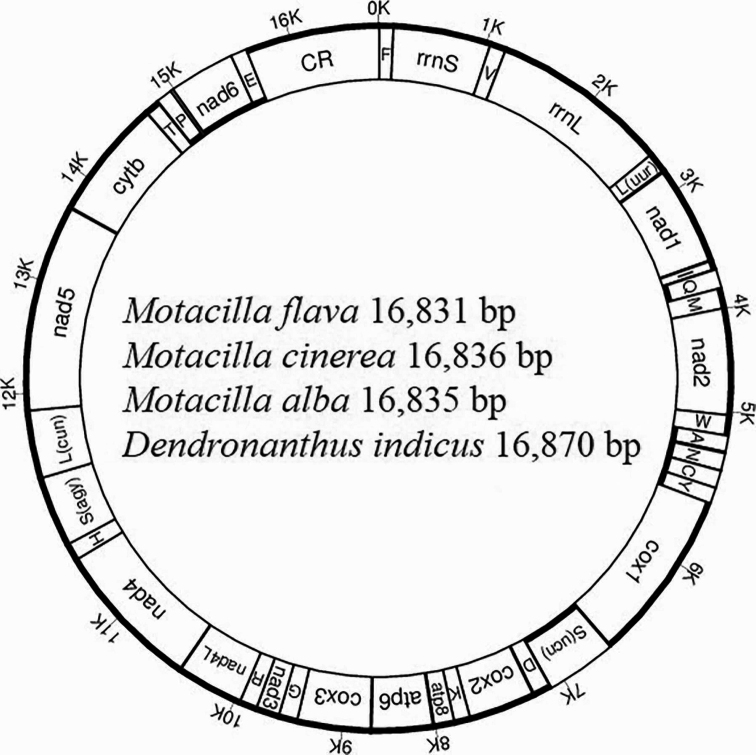
Gene map of four newly sequenced mitogenomes. Notes: tRNAs are abbreviated with a single letter; mitochondrial genes encoded by the J- and N-strands, indicated in bold, are located outside and inside of the circle, respectively.

An increasing number of avian mitogenome sequences are being generated with high-throughput sequencing technology (Morinha et al. 2016; Yang et al. 2018), facilitating the identification of mitogenomic characteristics such as gene order and base composition through the comparison of mitogenomes. However, the limited *Motacilla* mitogenomic sequences available from the GenBank database restricts the exploration of mitogenome features in this genus. For example, recent studies of *Motacilla* (Dong et al. 2016; Zhang et al. 2016; Harris et al. 2018; Gao et al. 2019) have focused on the phylogenetic relationships within this genus but have not conducted further comparative analyses among mitogenomes. In the present study, we obtained complete mitogenome sequences of *M.flava*, *M.cinerea*, *M.alba*, and *Dendronanthusindicus* Gmelin, 1789, performed comparative analyses and generated phylogenies (Subspecies differentiation was not discussed here). The new mitogenome data not only may help us understand the mitogenomic characteristics of *Motacilla* but also provide a basis for exploring phylogenetic relationships.

## ﻿Methods

### ﻿Specimen collection

Muscle samples were collected from the following species: *M.flava* (from China, Shaanxi Province, Hongjiannao in 2013); *M.cinerea* (from China, Shaanxi Province, Feng County in 2017); and *M.alba* and *D.indicus* (from China, Shaanxi Province, Lantian in 2018). All specimens of muscle samples were preserved in 100% ethanol and stored at -20 °C at the Shaanxi Institute of Zoology, Shaanxi Province, China.

### ﻿Mitogenome sequencing, assembly and annotation

The mitogenome of *M.flava* was sequenced by Genesky Biotechnologies Inc., Shanghai, China, using the Illumina HiSeq2000 platform, while those of *M.cinerea*, *M.alba* and *D.indicus* were sequenced at Biomarker Technologies Inc., Beijing, China, using the Illumina Xten platform and a 150 bp paired-end strategy. Genomic DNA was extracted using a DNeasy kit and fragmented using ultrasonic methods to prepare a small-inserted-fragment library. The library data were obtained via Bridge PCR and Illumina paired-end sequencing.

There were 15,149,744 paired-end raw reads of *M.flava*, of which 47,390 reads were used for mitogenome assembly, with average coverage of 417.1X. There were 20,702,440 paired-end raw reads of *M.cinerea*, with clean data 6.92 G. A total of 261,229 reads were used for mitogenome assembly, with average coverage of 2256.2X. There were 7,868,047 raw reads in *M.alba*, with 7,860,296 reads with clean data, and 8,430,436 raw reads of *D.indicus*, with 8,420,710 reads with clean data.

The raw data from *M.flava*, *M.cinerea* and *M.alba* were quality trimmed with CLC Genomics Workbench 9.5.2 (CLC bio, Aarhus, Denmark) using the default parameters. Mitogenome assembly was performed in MITOBIM 1.8 (Hahn et al. 2013), with *M.alba* (GenBank: NC029229) as a reference. The mitogenomic sequences of *D.indicus* were assembled using MitoZ 2.4 (Meng et al. 2019). Mitochondrial PCGs were identified using Geneious 11.1.3 (Kearse et al. 2012) by searching for open reading frames and employing the *M.alba* mitogenome (GenBank: NC029229) as a reference. Most tRNAs were identified using tRNAscan-SE 1.21 (Lowe and Eddy 1997), with secondary structures used as references. The remaining tRNAs, rRNAs and CRs were identified by comparison with other *Motacilla* species. Each mitochondrial gene was confirmed by alignment with the corresponding homologous genes from other *Motacilla* species available in GenBank. The secondary structures of rrnS and rrnL were generated using the mitogenomic rRNAs of *Remizconsobrinus* as a reference (Gao et al. 2013).

### ﻿Comparative analysis and phylogenetic reconstruction

The six mitogenomes (*M.flava*, *M.cinerea* and *M.alba* mitogenomes from collected specimens combined with *M.tschutschensis*, *M.alba* and *M.cinerea* genomes from GenBank) were used for comparative analysis. A mitogenome of *M.lugens* (KU246035/NC_029703) was excluded because this has been shown to represent a chimera (Sangster and Luksenburg 2021). The nucleotide compositions of the mitogenomes and different datasets were calculated using Geneious 11.1.3 (Kearse et al. 2012). Nucleotide bias was calculated using the formulas AT-skew = (A−T)/(A+T) and GC-skew = (G−C)/(G+C) (Perna and Kocher 1995). Relative synonymous codon usage (RSCU) was calculated with MEGA 11 (Tamura et al. 2021).

A total of 90 mitogenomes of Passeriformes were used to reconstruct phylogenetic relationships; the included mitogenomes came from 12 taxonomic families with *Aethopygagouldiae* (Nectariniidae) used as an outgroup (Suppl. material 7: Table S1). Each mitochondrial gene was aligned individually using MUSCLE in MEGA 11 (Tamura et al. 2021), starting with the alignment of PCGs to amino acid sequences. One mitogenomic dataset (mtDNA) was used for phylogenetic analysis, which included the nucleotide sequences of 13 PCGs, two rRNAs and 22 tRNAs, with a length of 15,722 bp. The best models of GTR+F+R5 for maximum likelihood (ML) analysis and GTR+F+I+G4 for Bayesian inference (BI) analysis were assessed in ModelFinder (Kalyaanamoorthy et al. 2017) using the Bayesian information criterion (BIC) in PhyloSuite 1.2.1 (Zhang et al. 2020). Phylogenetic relationships were analyzed using ML phylogenies with IQ-TREE 1.6.8 (Nguyen et al. 2015) with 1000 bootstrap replicates. The BI phylogeny was analysed with MrBayes 3.2.7 (Ronquist et al. 2012). Two independent runs with four simultaneous Markov chains were run for 5,000,000 generations and were sampled every 100 generations. The first 25% of generations were discarded as burn-in. The effective sample size (ESS) values were estimated in Tracer 1.7 (Rambaut et al. 2018), with ESS values > 200.

## ﻿Results and discussion

### ﻿Mitogenomic structure and organization

The obtained complete mitogenomes of *M.flava*, *M.cinerea*, *M.alba* and *D.indicus* ranged from 16,831 bp to 16,870 bp in length and each contained 37 genes and a noncoding region (CR) (Fig. 1). The complete mitogenome sequences were submitted to GenBank (MW929088–MW929091). Four gene arrangements have been identified among the Passeriformes mitogenomes sequenced to date (Caparroz et al. 2018; Mackiewicz et al. 2019). The gene order cytb-trnT-trnP-nad6-trnE-CR-trnF-rrnS is found in the mitogenomes of three *Motacilla* species and *D.indicus*, which is consistent with the order observed in most Passeriformes species (Mackiewicz et al. 2019). The major strand (J-strand) encodes 12 PCGs and two rRNAs as well as trnF, trnV, trnL(uur), trnI, trnM, trnW, trnD, trnK, trnG, trnR, the HSL cluster [trnH, trnS(agy), trnL(cun)] and trnT (Fig. 1). The lengths of the intergenic spacers range from 1–23 bp in the three *Motacilla* mitogenomes and 1–18 bp in *D.indicus*, with the longest intergenic spacer being located between the trnP and nad6 genes.

### ﻿Comparative analysis of *Motacilla* mitogenomes

The gene orders and nucleotide compositions of the six sampled *Motacilla* mitogenomes were generally similar. For instance, the A+T content ranges from 53.5% to 53.9%, which was slightly higher than the G+C contents. All mitogenomes showed a tendency toward A-skew and obvious C-skew (Suppl. material 8: Table S2), which was similar to findings in other birds (Kan et al. 2010; Eberhard and Wright 2016; Li et al. 2016a).

### ﻿Protein-coding genes

The A+T contents of the 13 PCGs excluding stop codons ranged from 52.4% to 52.9% in sampled *Motacilla* mitogenomes (Suppl. material 8: Table S2). The highest A+T content was found at the second codon position in all *Motacilla* mitogenomes. Obvious T-skew was recovered at the second codon position, while the most significant A-skew was found at the third codon position. The three codon positions showed diﬀerent degrees of C-skew, which was most obvious at the third codon position.

The first and last codons of the PCGs of *Motacilla* were compared (Suppl. material 9: Table S3). Twelve of the 13 PCGs started with an ATG codon, while nad3 started with ATT. The start codons were conserved in the six mitogenomes. The termination codons of the PCGs included TAA, TAG, AGG, AGA, TA and T, which are conserved among PCGs with the exception of cox2, cox3 and nad2. The incomplete T termination codons found in cox3 and nad4 have also been reported in other avian mitogenomes (Ma et al. 2014; Li et al. 2015; Eberhard and Wright 2016).

**Figure 2.**
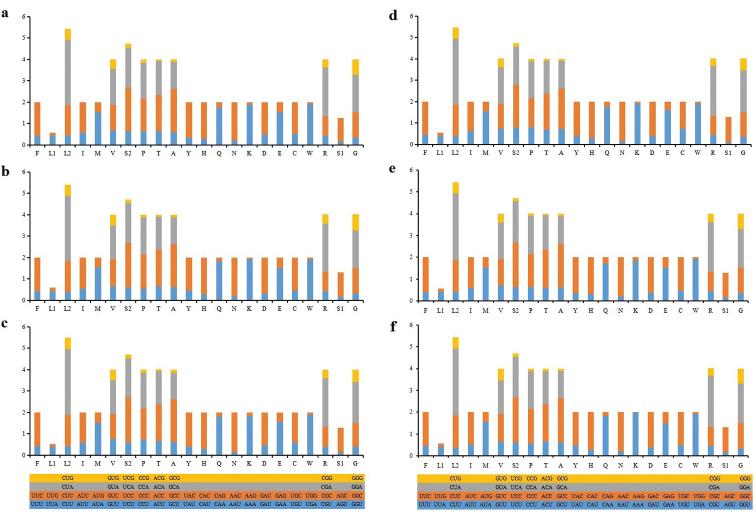
RSCU analysis of the PCGs of six mitogenomes from the genus *Motacilla*. Note **a***M.alba***b***M.cinerea***c***M.flava***d***M.tschutschensis***e***M.alba* (MN356232) **f***M.cinerea* (NC_027933).

RSCU analysis indicated that among all PCGs, codons including A or C at the third position were frequently overused relative to other synonymous codons (Fig. 2). The codon usage among *Motacilla* species was found to be conserved, with CUA (L), CGA (R) and UCC(S) representing the most frequently used codons.

### ﻿RNA genes

Similar to other avian mitogenomes, rrnS was found to be located between trnF and trnV, and rrnL was located between trnV and trnL(uur). The length of rrnS was 975 bp in *M.alba* (MN356232) and 973 bp in the other *Motacilla* mitogenomes, while the length of rrnL was 1595 bp in all *Motacilla* species. The A+T content was slightly greater than the G+C content in the rRNA genes, ranging from 52.2% to 52.3% in rrnS and 55.2% to 55.4% in rrnL, and both rRNA genes exhibited A-skew and C-skew (Suppl. material 8: Table S2).

The rrnS included three domains and 47 helices in *M.flava* (Suppl. material 2: Fig. S2), while rrnL included six domains and 60 helices (Suppl. material 3: Fig. S3). Most of the identified sequences and secondary structures were conserved compared with those of other *Motacilla* rRNAs. In addition, most of the stems of the rRNA secondary structures were similar to those found in other Passeriformes mitogenomes. For example, stems 21 and 47 of rrnS and 15 and 40 of rrnL were consistent with those found in *R.consobrinus* (Gao et al. 2013).

A total of eight tRNAs (trnQ, trnA, trnN, trnC, trnY, trnS(ucn), trnP and trnE) were located on the N-strand, while the remaining 14 tRNAs were located on the J-strand (Fig. 1). The lengths of the 22 tRNAs in each *Motacilla* species ranged from 66 to 75 bp. The A+T content ranged from 58.3% to 58.6% in the tRNAs, which exhibited A-skew and G-skew (Suppl. material 8: Table S2).

Twenty-one of the 22 tRNAs of *M.flava* were folded into a clover-leaf-like secondary structure, with the exception of trnS(agy), lacking a dihydrouridine (DHU) stem (Suppl. material 4: Fig. S4), which is considered to be a typical feature of metazoan mitogenomes (Wolstenholme 1992). Comparisons among *Motacilla* tRNAs showed that the most conserved tRNAs were trnL(UUR), trnM, trnW, trnA, trnC, trnH, trnL(CUN), trnT, trnP and trnE (Suppl. material 4: Fig. S4), which contained the same nucleotides. Some mismatched base pairs found in *Motacilla* were similar to those observed in some other Passeriformes species (*Pyrgilaudaruficollis*, Ma et al. 2014; *R.consobrinus*, Gao et al. 2013), such as the C-C pair located in the acceptor stem of trnL(uur) and the anticodon stem of trnG, A-A in the TφC stem of trnD, and U-U in the anticodon stem of trnG.

### ﻿Control region

The CR was located between the trnE and trnF genes and were 1243–1250 bp in length. The average A+T content was 56.2% among all sampled *Motacilla* mitogenomes, which was slightly higher than that of G+C. The CRs showed a tendency toward T-skew and C-skew (Suppl. material 8: Table S2), with C-skew being more obvious. This C-skew was consistent with findings in other reported avian CRs (e.g., Huang et al. 2017).

The CR regulates the replication of the H strand and the transcription of all mitochondrial genes (Clayton 1992) and can be divided into three domains: extended termination-associated sequence (ETAS) domain I, central conserved domain II, and conserved sequence block (CSB) domain III (Sbisà et al. 1997; Randi and Lucchini 1998; Ruokonen and Kvist 2002). Among the three domains of the CR, domain I showed slight A-skew and obvious C-skew, domain II showed a tendency toward T-skew and C-skew, and domain III exhibited A-skew and a highly significant C-skew (Suppl. material 8: Table S2).

The proportions of variable sites among the three domains were 3.6%, 2.4% and 9.0%, respectively. Thus, most variation was found in domain III, similar to the findings of previous studies (Ruokonen and Kvist 2002; Huang et al. 2017). A poly-C sequence was found near the 5’ end of CR domain I in *M.flava*, with a sequence of CCCCCCCCCCTTCCCCCCCC, and this sequence was relatively conserved in the sampled mitogenome CRs (Suppl. material 5: Fig. S5). Within the *M.flava* CR sequence, boxes F, E, D, C, B and a bird similarity box in domain II were identified. The F, E, D and C boxes were similar to those found in other avian mitogenomes (Suppl. material 5: Fig. S5; Huang et al. 2017). Among these boxes, the F-box, bird similarity box and B-box were fully conserved among sampled mitogenomic sequences. Domain III contained CSB1, whose sequence was similar to that found in other birds (Huang et al. 2017). However, it was difficult to identify sequences corresponding to O_H_, CSB2, CSB3 and bidirectional LSP/HSP promoters found in other birds (Li et al. 2015), which might play important roles in mitogenome replication. Furthermore, tandem repeat sequences in CRs are found in many avian mitogenomes (Yang et al. 2018). However, none of the sampled *Motacilla* CRs contained tandem repeats.

### ﻿Phylogenetic analysis

**Figure 3.**
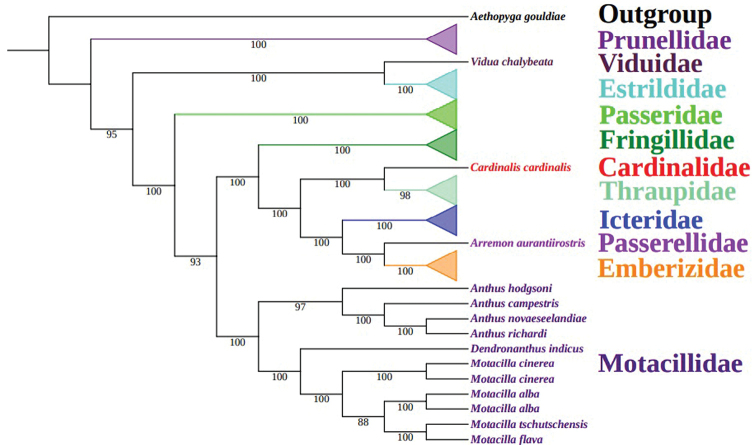
Phylogenetic results based on the maximum likelihood method using the mtDNA dataset.

The ML and BI phylogenetic trees were reconstructed using the mtDNA dataset, showing consistent topological results among Motacillidae (Fig. 3 and Suppl. material 6: Fig. S6). The analyses supported the monophyly of Motacillidae with 100% bootstrap support and posterior probabilities of 1.0. Among the three sampled genera among Motacillidae (*Anthus*, *Dendronanthus* and *Motacilla*), *Anthus* was sister to *D.indicus* and *Motacilla*. The monophyly of *Motacilla* was also recovered, with *D.indicus* forming a sister group with *Motacilla*.

Within *Motacilla*, the following phylogenetic relationships were recovered: (((*M.flava*+*M.tschutschensis*)+*M.alba*)+*M.cinerea*), similar to previous studies (Suppl. material 1: Fig. S1A; Dong et al. 2016; Suppl. material 1: Fig. S1D; Gao et al. 2019). *Motacillacinerea* was in the basal position within *Motacilla*, and *M.flava* showed a closer relationship with *M.tschutschensis*. However, *M.alba* showed a closer relationship with *M.cinerea* (Suppl. material 1: Fig. S1B; Zhang et al. 2016), while *M.cinerea* presented a closer phylogenetic relationship with *M.flava* (Suppl. material 1: Fig. S1C; Harris et al. 2018). These differences might be due to the different data types, dataset sizes and sampling strategies involved. For example, the phylogenetic tree topologies obtained from the complete mitogenome are not identical to those resulting from individual mitochondrial genes in some avian taxa (Campillo et al. 2019). In addition, the phylogenetic relationships recovered from nuclear segment datasets are inconsistent with those recovered from mitogenomes in some aves (Li et al. 2016a; Campillo et al. 2019). Therefore, our results indicate that further studies are needed to address the phylogenetic relationships within *Motacilla* by adding more sampling and some nuclear data.

## ﻿Conclusions

The complete mitogenomes of *Motacillaflava*, *M.cinerea*, *M.alba* and *Dendronanthusindicus* were sequenced and were shown to present the typical genome organization and gene order found in other Passeriformes mitogenomes. We focused on comparative analyses of the six mitogenomes to identify the mitogenomic characteristics of the genus *Motacilla*, such as the base composition, codon usage and RNA secondary structures. The complete mitogenomes showed a tendency toward A-skew and C-skew. Most PCGs start with typical ATG codons and terminated with TAA codons. All tRNAs could be folded into classic clover-leaf structures except for trnS(agy), which lacked a DHU arm. In addition, 90 mitogenomes of Passeriformes were used to build the tree of phylogenetic relationships. The phylogenetic tree supported the monophyly of Motacillidae. Within *Motacilla*, the phylogenetic topology of (((*M.flava*+*M.tschutschensis*)+*M.alba*)+*M.cinerea*) was recovered.

## Supplementary materials

Supplementary material 1 Figure S1 Data type Image. Explanation note Phylogenetic hypotheses of previous studies. a: Dong et al. (2016); b: Zhang et al. (2016); c: Harris et al. (2018); d: Gao et al. (2019). https://binary.pensoft.net/file/708201This dataset is made available under the Open Database License (http://opendatacommons.org/licenses/odbl/1.0/). The Open Database License (ODbL) is a license agreement intended to allow users to freely share, modify, and use this Dataset while maintaining this same freedom for others, provided that the original source and author(s) are credited.Chao Yang, Xiaojuan Du, Yuxin Liu, Hao Yuan, Qingxiong Wang, Xiang Hou, Huisheng Gong, Yan Wang, Yuan Huang, Xuejuan Li, Haiyan Ye

Supplementary material 2 Figure S2 Data type Image. Explanation note Secondary structures of rrnS of *M.flava*. Note: differences within the six mitogenomes from the genus *Motacilla* are indicated by filled grey circles. https://binary.pensoft.net/file/708202This dataset is made available under the Open Database License (http://opendatacommons.org/licenses/odbl/1.0/). The Open Database License (ODbL) is a license agreement intended to allow users to freely share, modify, and use this Dataset while maintaining this same freedom for others, provided that the original source and author(s) are credited.Chao Yang, Xiaojuan Du, Yuxin Liu, Hao Yuan, Qingxiong Wang, Xiang Hou, Huisheng Gong, Yan Wang, Yuan Huang, Xuejuan Li, Haiyan Ye

Supplementary material 3 Figure S3 Data type Image. Explanation note Secondary structures of rrnL of *M.flava*. Note: differences within the six mitogenomes from the genus *Motacilla* are indicated by filled grey circles. https://binary.pensoft.net/file/708203This dataset is made available under the Open Database License (http://opendatacommons.org/licenses/odbl/1.0/). The Open Database License (ODbL) is a license agreement intended to allow users to freely share, modify, and use this Dataset while maintaining this same freedom for others, provided that the original source and author(s) are credited.Chao Yang, Xiaojuan Du, Yuxin Liu, Hao Yuan, Qingxiong Wang, Xiang Hou, Huisheng Gong, Yan Wang, Yuan Huang, Xuejuan Li, Haiyan Ye

Supplementary material 4 Figure S4 Data type Image. Explanation note Secondary structures of tRNAs of *M.flava*. Note: differences within the six mitogenomes from the genus *Motacilla* are indicated by filled grey circles. https://binary.pensoft.net/file/708204This dataset is made available under the Open Database License (http://opendatacommons.org/licenses/odbl/1.0/). The Open Database License (ODbL) is a license agreement intended to allow users to freely share, modify, and use this Dataset while maintaining this same freedom for others, provided that the original source and author(s) are credited.Chao Yang, Xiaojuan Du, Yuxin Liu, Hao Yuan, Qingxiong Wang, Xiang Hou, Huisheng Gong, Yan Wang, Yuan Huang, Xuejuan Li, Haiyan Ye

Supplementary material 5 Figure S5 Data type Image. Explanation note Control region structures of *M.flava*. Note: differences within the six mitogenomes from the genus *Motacilla* are indicated with filled circles. https://binary.pensoft.net/file/708205This dataset is made available under the Open Database License (http://opendatacommons.org/licenses/odbl/1.0/). The Open Database License (ODbL) is a license agreement intended to allow users to freely share, modify, and use this Dataset while maintaining this same freedom for others, provided that the original source and author(s) are credited.Chao Yang, Xiaojuan Du, Yuxin Liu, Hao Yuan, Qingxiong Wang, Xiang Hou, Huisheng Gong, Yan Wang, Yuan Huang, Xuejuan Li, Haiyan Ye

Supplementary material 6 Figure S6 Data type Image. Explanation note Maximum likelihood and Bayesian inference phylogenetic results based on the mtDNA dataset, corresponding to Fig. 3. Note: a: ML tree, b: BI tree. https://binary.pensoft.net/file/708206This dataset is made available under the Open Database License (http://opendatacommons.org/licenses/odbl/1.0/). The Open Database License (ODbL) is a license agreement intended to allow users to freely share, modify, and use this Dataset while maintaining this same freedom for others, provided that the original source and author(s) are credited.Chao Yang, Xiaojuan Du, Yuxin Liu, Hao Yuan, Qingxiong Wang, Xiang Hou, Huisheng Gong, Yan Wang, Yuan Huang, Xuejuan Li, Haiyan Ye

Supplementary material 7 Table S1 Data type Doc file. Explanation note Mitogenome sequences employed for reconstructing phylogenetic trees. https://binary.pensoft.net/file/708207This dataset is made available under the Open Database License (http://opendatacommons.org/licenses/odbl/1.0/). The Open Database License (ODbL) is a license agreement intended to allow users to freely share, modify, and use this Dataset while maintaining this same freedom for others, provided that the original source and author(s) are credited.Chao Yang, Xiaojuan Du, Yuxin Liu, Hao Yuan, Qingxiong Wang, Xiang Hou, Huisheng Gong, Yan Wang, Yuan Huang, Xuejuan Li, Haiyan Ye

Supplementary material 8 Table S2 Data type Doc file. Explanation note Nucleotide composition and bias of six mitogenomes of the genus *Motacilla*. Notes: Stop codons of protein-coding genes were excluded; AT-skew=[A-T]/[A+T], GC-skew=[G-C]/[G+C]. The sequenced mitogenome species in this study are shown in the bold format. https://binary.pensoft.net/file/708208This dataset is made available under the Open Database License (http://opendatacommons.org/licenses/odbl/1.0/). The Open Database License (ODbL) is a license agreement intended to allow users to freely share, modify, and use this Dataset while maintaining this same freedom for others, provided that the original source and author(s) are credited.Chao Yang, Xiaojuan Du, Yuxin Liu, Hao Yuan, Qingxiong Wang, Xiang Hou, Huisheng Gong, Yan Wang, Yuan Huang, Xuejuan Li, Haiyan Ye

Supplementary material 9 Table S3 Data type Doc file. Explanation note Initial and terminal codons of protein-coding genes in the six mitogenomes from the genus *Motacilla*. Notes: The sequenced mitogenome species in this study are shown in the bold format. https://binary.pensoft.net/file/708209This dataset is made available under the Open Database License (http://opendatacommons.org/licenses/odbl/1.0/). The Open Database License (ODbL) is a license agreement intended to allow users to freely share, modify, and use this Dataset while maintaining this same freedom for others, provided that the original source and author(s) are credited.Chao Yang, Xiaojuan Du, Yuxin Liu, Hao Yuan, Qingxiong Wang, Xiang Hou, Huisheng Gong, Yan Wang, Yuan Huang, Xuejuan Li, Haiyan Ye
