# Supplementary figures and images for: ﻿Comparative mitogenomics of the genus Motacilla (Aves, Passeriformes) and its phylogenetic implications
Source: Zookeys. 2022 Jul 1;1109:49–65. doi: 10.3897/zookeys.1109.81125 (PMC9848870; doi:10.3897/zookeys.1109.81125)

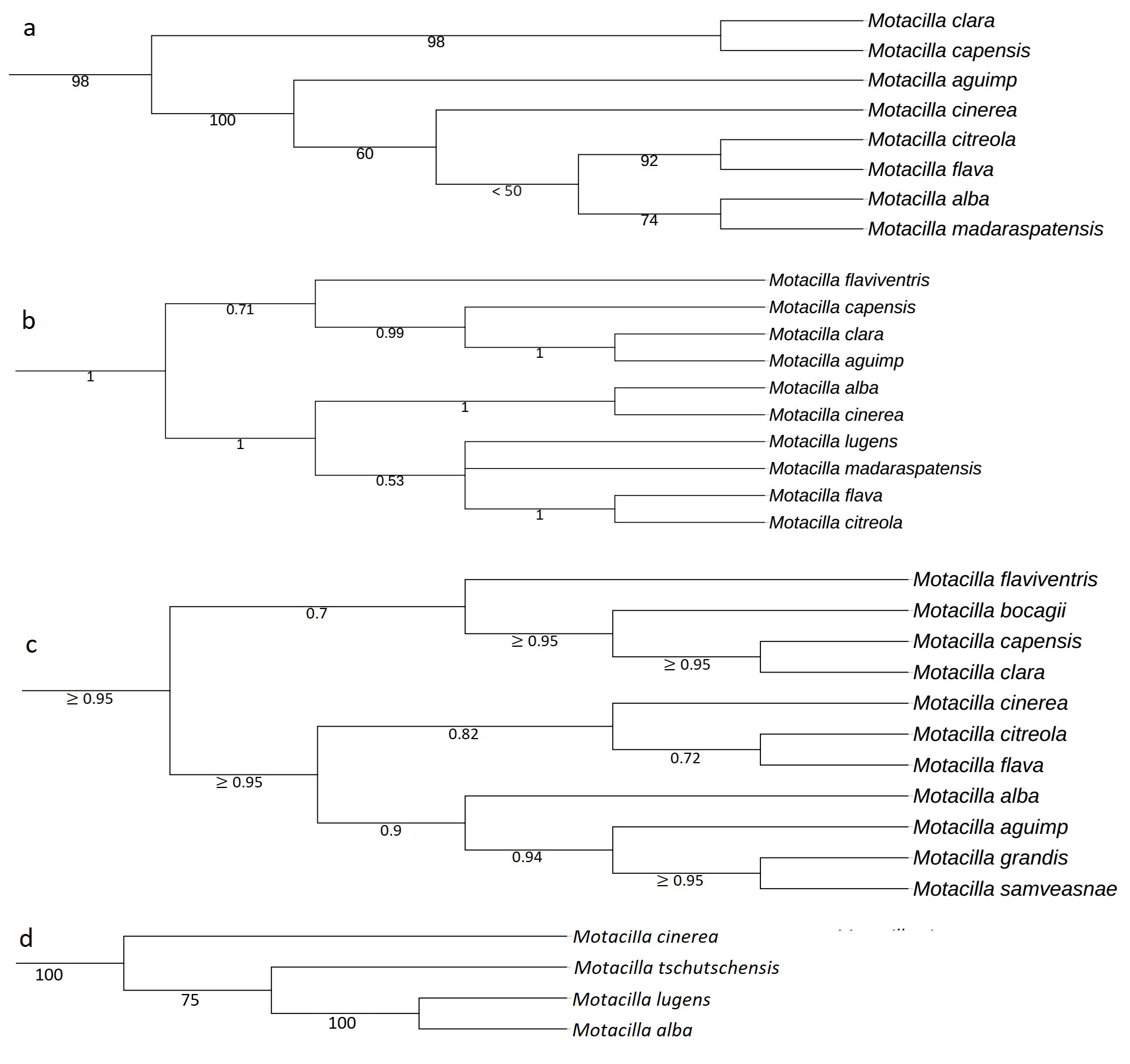

Supplement: Supplementary material 1 — Figure S1 [file zookeys-1109-049_article-81125__-s001.jpg]

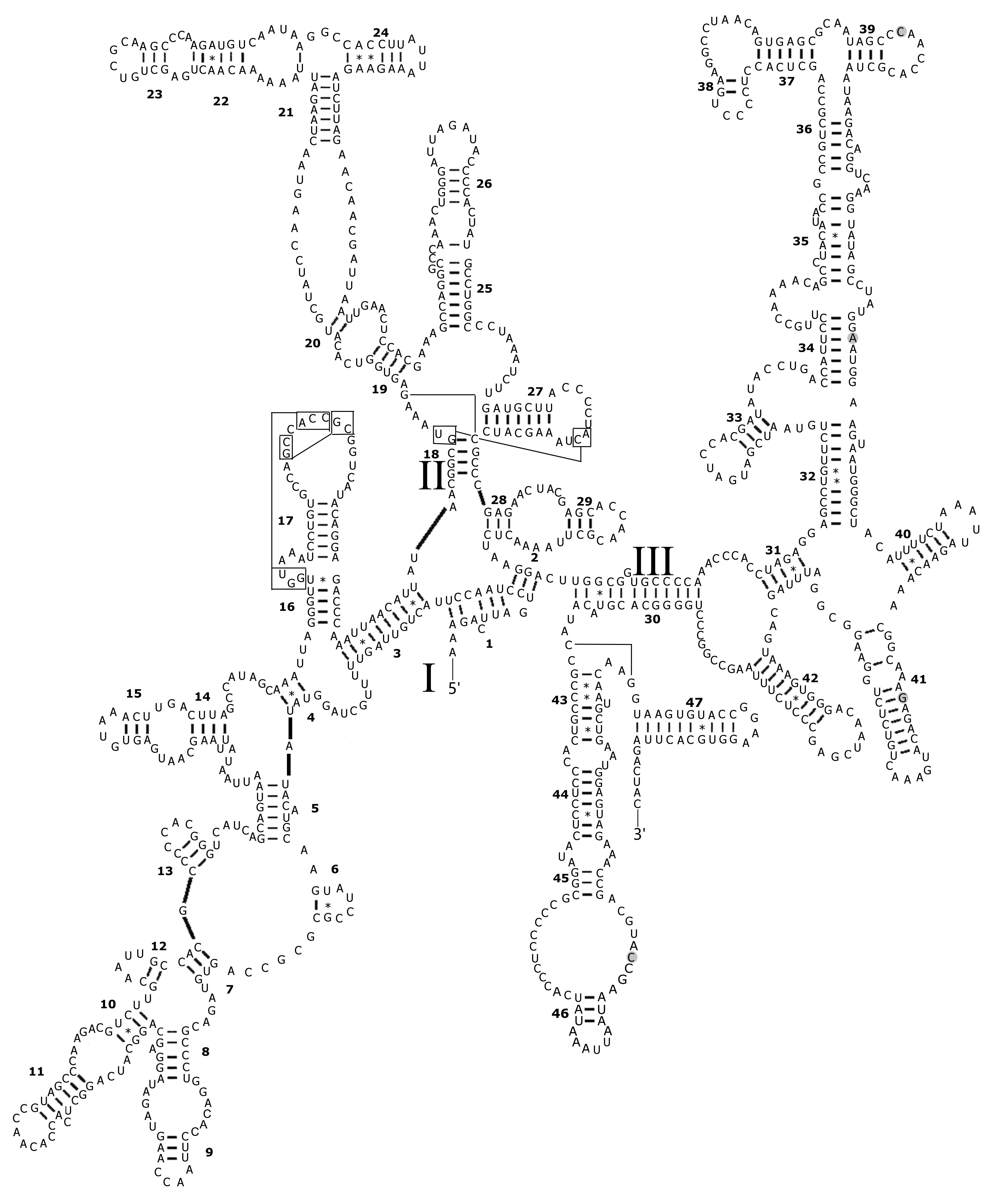

Supplement: Supplementary material 2 — Figure S2 [file zookeys-1109-049_article-81125__-s002.jpg]

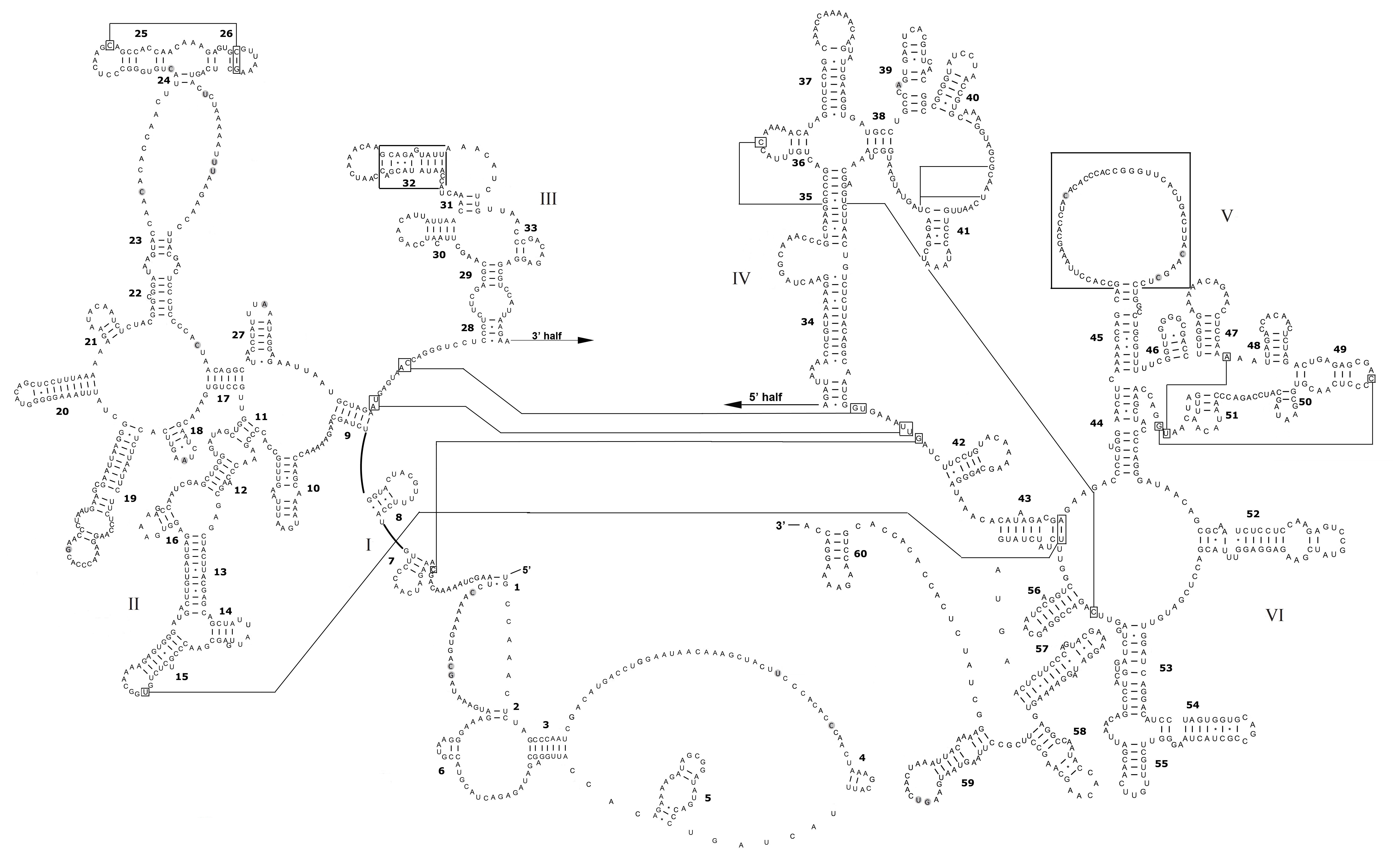

Supplement: Supplementary material 3 — Figure S3 [file zookeys-1109-049_article-81125__-s003.jpg]

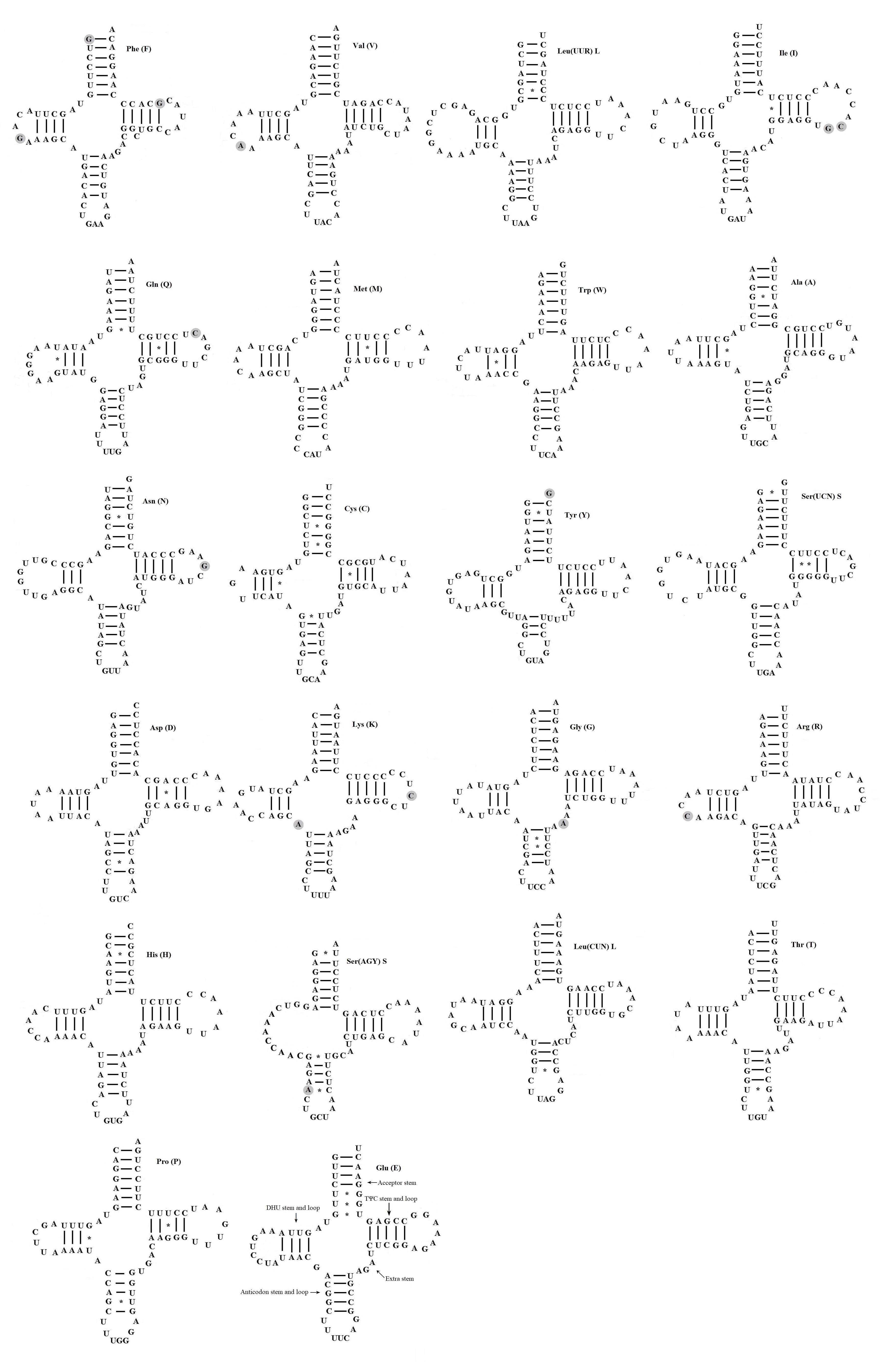

Supplement: Supplementary material 4 — Figure S4 [file zookeys-1109-049_article-81125__-s004.jpg]

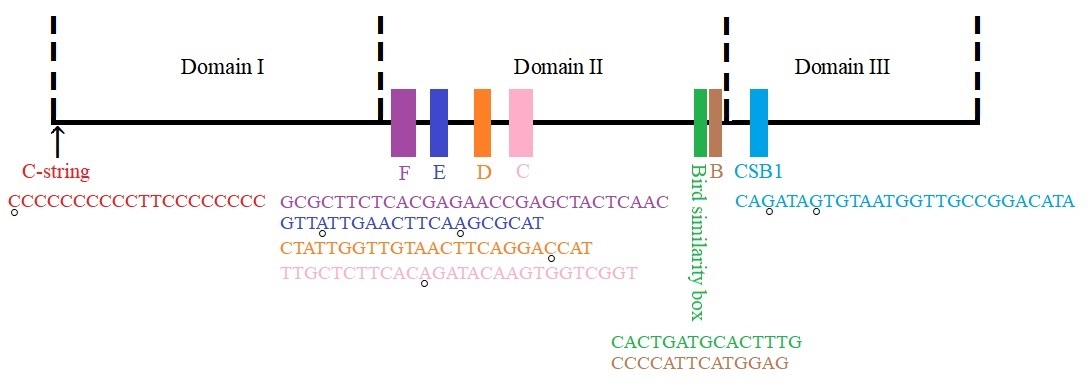

Supplement: Supplementary material 5 — Figure S5 [file zookeys-1109-049_article-81125__-s005.jpg]

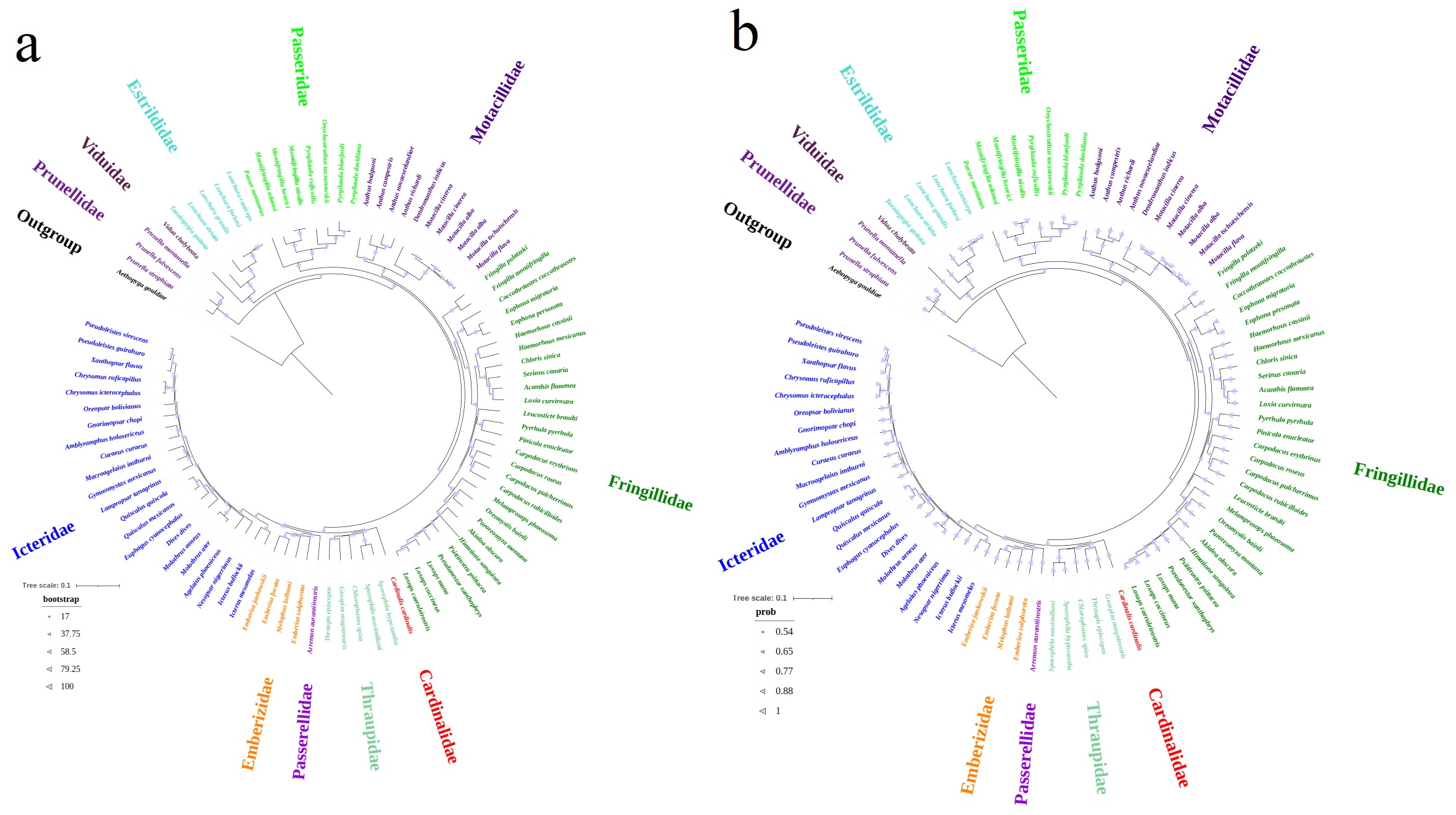

Supplement: Supplementary material 6 — Figure S6 [file zookeys-1109-049_article-81125__-s006.jpg]
