# Supplementary material for: ﻿Comparative mitogenomics of the genus Motacilla (Aves, Passeriformes) and its phylogenetic implications
Source: Zookeys. 2022 Jul 1;1109:49–65. doi: 10.3897/zookeys.1109.81125 (PMC9848870; doi:10.3897/zookeys.1109.81125)
Supplement: Supplementary material 7 — Table S1 [file zookeys-1109-049_article-81125__-s007.doc]

**Table S1.** The mitogenome sequences employed for reconstructing phylogenetic trees.

| Family | Species | GenBank accession no. |
| --- | --- | --- |
| Cardinalidae | *Cardinalis cardinalis* | MH700631 |
| Emberizidae | *Emberiza fucata* | NC_033338 |
| Emberizidae | *Emberiza jankowskii* | NC_027251 |
| Emberizidae | *Emberiza sulphurata* | KY419885 |
| Emberizidae | *Melophus lathami* | NC_031845 |
| Estrildidae | *Lonchura caniceps* | NC_036397 |
| Estrildidae | *Lonchura forbesi* | NC_036400 |
| Estrildidae | *Lonchura grandis* | NC_036403 |
| Estrildidae | *Lonchura striata* | NC_029475 |
| Estrildidae | *Taeniopygia guttata* | DQ453515 |
| Fringillidae | *Acanthis flammea* | NC_027285 |
| Fringillidae | *Akialoa obscura* | NC_031349 |
| Fringillidae | *Carpodacus rubicilloides* | NC_040975 |
| Fringillidae | *Carpodacus erythrinus* | MN122832 |
| Fringillidae | *Carpodacus pulcherrimus* | NC_051538 |
| Fringillidae | *Carpodacus roseus* | KM078779 |
| Fringillidae | *Chloris sinica* | MH102388 |
| Fringillidae | *Coccothraustes coccothraustes* | KM078789 |
| Fringillidae | *Eophona migratoria* | NC_031374 |
| Fringillidae | *Eophona personata* | KX812499 |
| Fringillidae | *Fringilla montifringilla* | NC_024048 |
| Fringillidae | *Fringilla polatzeki* | NC_031157 |
| Fringillidae | *Haemorhous cassinii* | KM078786 |
| Fringillidae | *Haemorhous mexicanus* | KM078782 |
| Fringillidae | *Himatione sanguinea* | NC_025602 |
| Fringillidae | *Leucosticte brandti* | NC_025604 |
| Fringillidae | *Loxia curvirostra* | NC_025623 |
| Fringillidae | *Loxops caeruleirostris* | NC_025605 |
| Fringillidae | *Loxops coccineus* | NC_025612 |
| Fringillidae | *Loxops mana* | NC_025598 |
| Fringillidae | *Melamprosops phaeosoma* | NC_025617 |
| Fringillidae | *Oreomystis bairdi* | NC_025628 |
| Fringillidae | *Paroreomyza montana* | NC_025601 |
| Fringillidae | *Pinicola enucleator* | NC_025609 |
| Fringillidae | *Pseudonestor xanthophrys* | NC_025630 |
| Fringillidae | *Psittirostra psittacea* | NC_031353 |
| Fringillidae | *Pyrrhula pyrrhula* | NC_025625 |
| Fringillidae | *Serinus canaria* | NC_037521 |
| Icteridae | *Agelaius phoeniceus* | MN356439 |
| Icteridae | *Amblyramphus holosericeus* | NC_018802 |
| Icteridae | *Chrysomus icterocephalus* | NC_018799 |
| Icteridae | *Chrysomus ruficapillus* | NC_018796 |
| Icteridae | *Curaeus curaeus* | NC_018808 |
| Icteridae | *Dives dives* | NC_018800 |
| Icteridae | *Euphagus cyanocephalus* | NC_018827 |
| Icteridae | *Gnorimopsar chopi* | NC_018795 |
| Icteridae | *Gymnomystax mexicanus* | NC_018812 |
| Icteridae | *Icterus bullockii* | FJ236287 |
| Icteridae | *Icterus mesomelas* | JX516068 |
| Icteridae | *Lampropsar tanagrinus* | JX516057 |
| Icteridae | *Macroagelaius imthurni* | NC_018810 |
| Icteridae | *Molothrus aeneus* | NC_018806 |
| Icteridae | *Molothrus ater* | NC_051468 |
| Icteridae | *Nesopsar nigerrimus* | NC_018794 |
| Icteridae | *Oreopsar bolivianus* | NC_018797 |
| Icteridae | *Pseudoleistes guirahuro* | NC_018809 |
| Icteridae | *Pseudoleistes virescens* | NC_018805 |
| Icteridae | *Quiscalus mexicanus* | NC_051021 |
| Icteridae | *Quiscalus quiscula* | NC_018803 |
| Icteridae | *Xanthopsar flavus* | NC_018804 |
| Motacillidae | *Anthus novaeseelandiae* | NC_029137 |
| Motacillidae | *Anthus campestris* | MT410931 |
| Motacillidae | *Anthus hodgsoni* | KX189345 |
| Motacillidae | *Anthus richardi* | NC_041109 |
| Motacillidae | *Motacilla alba* | MW929091 |
| Motacillidae | *Motacilla alba* | MN356232 |
| Motacillidae | *Motacilla cinerea* | MW929089 |
| Motacillidae | *Motacilla cinerea* | NC_027933 |
| Motacillidae | *Motacilla flava* | MW929090 |
| Motacillidae | *Motacilla tschutschensis* | MN217252 |
| Motacillidae | *Dendronanthus indicus* | MW929088 |
| Nectariniidae | *Aethopyga gouldiae* | NC_027241 |
| Passerellidae | *Arremon aurantiirostris* | NC_027731 |
| Passeridae | *Montifringilla adamsi* | NC_025913 |
| Passeridae | *Montifringilla henrici* | MH049432 |
| Passeridae | *Montifringilla nivalis* | KJ148628 |
| Passeridae | *Onychostruthus taczanowskii* | KJ148631 |
| Passeridae | *Passer montanus* | NC_024821 |
| Passeridae | *Pyrgilauda blanfordi* | NC_025912 |
| Passeridae | *Pyrgilauda davidiana* | KJ148632 |
| Passeridae | *Pyrgilauda ruficollis* | KC836121 |
| Prunellidae | *Prunella fulvescens* | NC_035747 |
| Prunellidae | *Prunella montanella* | NC_027284 |
| Prunellidae | *Prunella strophiata* | NC_031819 |
| Thraupidae | *Chlorophanes spiza* | NC_025606 |
| Thraupidae | *Geospiza magnirostris* | NC_039770 |
| Thraupidae | *Sporophila hypoxantha* | NC_051465 |
| Thraupidae | *Sporophila maximiliani* | NC_035673 |
| Thraupidae | *Thraupis episcopus* | NC_025596 |
| Viduidae | *Vidua chalybeata* | NC_000880 |
