# Supplementary material for: ﻿Comparative mitogenomics of the genus Motacilla (Aves, Passeriformes) and its phylogenetic implications
Source: Zookeys. 2022 Jul 1;1109:49–65. doi: 10.3897/zookeys.1109.81125 (PMC9848870; doi:10.3897/zookeys.1109.81125)
Supplement: Supplementary material 8 — Table S2 [file zookeys-1109-049_article-81125__-s008.doc]

**Table S2.** Nucleotide composition and bias of six mitogenomes of the genus *Motacilla*.

| Feature | A+T content (%) | | | | | | AT-skew | | | | | | GC-skew | | | | | |
| --- | --- | --- | --- | --- | --- | --- | --- | --- | --- | --- | --- | --- | --- | --- | --- | --- | --- | --- |
|  | ***M. fla*** | ***M. cin*** | ***M. alb*** | *M. tsc* | *M. cin* | *M. alb* | ***M. fla*** | ***M. cin*** | ***M. alb*** | *M. tsc* | *M. cin* | *M. alb* | ***M. fla*** | ***M. cin*** | ***M. alb*** | *M. tsc* | *M. cin* | *M. alb* |
| Mitogenome | 53.6 | 53.5 | 53.7 | 53.9 | 53.5 | 53.7 | 0.1231 | 0.1252 | 0.1210 | 0.1206 | 0.1252 | 0.1248 | -0.3707 | -0.3707 | -0.3693 | -0.3709 | -0.3720 | -0.3707 |
| PCG | 52.5 | 52.4 | 52.6 | 52.9 | 52.5 | 52.6 | 0.0705 | 0.0725 | 0.0722 | 0.0662 | 0.0743 | 0.0722 | -0.3924 | -0.3908 | -0.3911 | -0.3928 | -0.3937 | -0.3924 |
| PCG-1st | 47.4 | 47.5 | 47.4 | 47.4 | 47.4 | 47.4 | 0.1603 | 0.1537 | 0.1561 | 0.1561 | 0.1561 | 0.1561 | -0.1044 | -0.0992 | -0.0989 | -0.1006 | -0.1010 | -0.0989 |
| PCG-2nd | 58.6 | 58.5 | 58.7 | 58.5 | 58.5 | 58.7 | -0.3823 | -0.3846 | -0.3833 | -0.3846 | -0.3846 | -0.3833 | -0.3720 | -0.3720 | -0.3753 | -0.3735 | -0.3735 | -0.3720 |
| PCG-3rd | 51.6 | 51.4 | 51.8 | 52.9 | 51.4 | 51.8 | 0.5000 | 0.5175 | 0.5097 | 0.4858 | 0.5214 | 0.5135 | -0.7190 | -0.7243 | -0.7220 | -0.7373 | -0.7248 | -0.7303 |
| rRNA | 54 | 54.2 | 54.3 | 54.1 | 54.1 | 54.1 | 0.2259 | 0.2251 | 0.2265 | 0.2237 | 0.2237 | 0.2274 | -0.1130 | -0.1092 | -0.1160 | -0.1111 | -0.1092 | -0.1179 |
| rrnS | 52.2 | 52.3 | 52.3 | 52.2 | 52.3 | 52.3 | 0.2008 | 0.1945 | 0.2024 | 0.2008 | 0.1931 | 0.2046 | -0.1226 | -0.1164 | -0.1250 | -0.1226 | -0.1153 | -0.1279 |
| rrnL | 55.2 | 55.3 | 55.4 | 55.2 | 55.3 | 55.2 | 0.2409 | 0.2426 | 0.2398 | 0.2395 | 0.2405 | 0.2428 | -0.1077 | -0.1024 | -0.1111 | -0.1064 | -0.1007 | -0.1116 |
| tRNA | 58.3 | 58.6 | 58.5 | 58.4 | 58.5 | 58.6 | 0.0532 | 0.0512 | 0.0530 | 0.0479 | 0.0496 | 0.0546 | 0.0072 | 0.0097 | 0.0072 | 0.0048 | 0.0072 | 0.0048 |
| CR | 56.2 | 55.6 | 56.4 | 56.6 | 55.7 | 56.6 | -0.0214 | -0.0288 | -0.0248 | -0.0177 | -0.0269 | -0.0247 | -0.3653 | -0.3604 | -0.3532 | -0.3733 | -0.3544 | -0.3580 |
| CR-domainI | 49.9 | 49 | 50 | 50.4 | 49 | 50.4 | 0.0581 | 0.0694 | 0.0600 | 0.0675 | 0.0694 | 0.0635 | -0.3533 | -0.3464 | -0.3480 | -0.3535 | -0.3438 | -0.3521 |
| CR-domainII | 54.2 | 53.7 | 54.2 | 53.7 | 53.7 | 54.2 | -0.1513 | -0.1695 | -0.1587 | -0.1620 | -0.1508 | -0.1587 | -0.1921 | -0.1818 | -0.1834 | -0.1922 | -0.1922 | -0.1834 |
| CR-domainIII | 64.7 | 64 | 65.1 | 65.5 | 64.3 | 65.3 | 0.0263 | 0.0188 | 0.0230 | 0.0321 | 0.0078 | 0.0199 | -0.6045 | -0.6156 | -0.5874 | -0.6406 | -0.5854 | -0.5965 |

Notes: Stop codons of protein-coding genes were excluded; AT-skew=[A-T]/[A+T], GC-skew=[G-C]/[G+C]; The sequenced mitogenome species in this study are shown in the bold format.
