# Supplementary material for: ﻿Comparative mitogenomics of the genus Motacilla (Aves, Passeriformes) and its phylogenetic implications
Source: Zookeys. 2022 Jul 1;1109:49–65. doi: 10.3897/zookeys.1109.81125 (PMC9848870; doi:10.3897/zookeys.1109.81125)
Supplement: Supplementary material 9 — Table S3 [file zookeys-1109-049_article-81125__-s009.doc]

**Table S3.** Initial and terminal codons for protein-coding genes of six mitogenomes of the genus *Motacilla*.

| Species | Gene | | | | | | | | | | | | |
| --- | --- | --- | --- | --- | --- | --- | --- | --- | --- | --- | --- | --- | --- |
|  | atp6 | atp8 | cox1 | cox2 | cox3 | cytb | nad1 | nad2 | nad3 | nad4 | nad4L | nad5 | nad6 |
| ***M. flava*** | ATG/TAA | ATG/TAA | ATG/AGG | ATG/TAA | ATG/T | ATG/TAA | ATG/AGA | ATG/TAA | ATT/TAA | ATG/T | ATG/TAA | ATG/AGA | ATG/TAG |
| ***M. cinerea*** | ATG/TAA | ATG/TAA | ATG/AGG | ATG/TAA | ATG/T | ATG/TAA | ATG/AGA | ATG/TAA | ATT/TAA | ATG/T | ATG/TAA | ATG/AGA | ATG/TAG |
| ***M. alba*** | ATG/TAA | ATG/TAA | ATG/AGG | ATG/TAA | ATG/T | ATG/TAA | ATG/AGA | ATG/TAA | ATT/TAA | ATG/T | ATG/TAA | ATG/AGA | ATG/TAG |
| *M. tschutschensis* | ATG/TAA | ATG/TAA | ATG/AGG | ATG/TA | ATG/T | ATG/TAA | ATG/AGA | ATG/TA | ATT/TAA | ATG/T | ATG/TAA | ATG/AGA | ATG/TAG |
| *M. cinerea* | ATG/TAA | ATG/TAA | ATG/AGG | ATG/TAA | ATG/T | ATG/TAA | ATG/AGA | ATG/TA | ATT/TAA | ATG/T | ATG/TAA | ATG/AGA | ATG/TAG |
| *M. alba* | ATG/TAA | ATG/TAA | ATG/AGG | ATG/TAA | ATG/TA | ATG/TAA | ATG/AGA | ATG/TA | ATT/TAA | ATG/T | ATG/TAA | ATG/AGA | ATG/TAG |

Notes: The sequenced mitogenome species in this study are shown in the bold format.
